# Huge Retroperitoneal Dermoid: A Presentation

**DOI:** 10.1055/s-0039-1697630

**Published:** 2019-10-09

**Authors:** Tanweerul Huda, Mahendra Pratap Singh

**Affiliations:** 1 Department of General Surgery, AIIMS, Bhopal, Madhya Pradesh, India

**Keywords:** dermoid, retroperitoneal cysts, teratomas, cystic mass, retrocaval mass

## Abstract

Teratoma can be defined as a true neoplasm that contains tissues that either are foreign to the primary site of origin or are histologically diverse and represent more than one of the embryonic germ layers. A 20-year-old female patient presented with chief complaints of swelling in the right upper abdomen since childhood and back pain for the past 4 years. Per abdomen, examination revealed a lump of around 15 cm in size in the right hypochondrial region extending to the epigastric region. Contrast-enhanced computed tomography abdomen revealed a 14.3 × 14.1 × 17.4 cm well-defined heterogeneously hypoattenuating nonenhancing complex cystic mass with focal areas of calcifications and fat attenuation in retroperitoneum. The patient was taken up for exploratory laparotomy and a tumor was found in the retroperitoneum, retrocavally and was excised with due care. Histopathological examination features were suggestive of mature cystic teratoma. The postoperative stay was uneventful.

Teratoma can be defined as a true neoplasm that contains tissues that either are foreign to the primary site of origin or are histologically diverse and represent more than one of the embryonic germ layers. 1 They often contain both cystic and solid components. Teratomas are more common in children than in adults, but when found in adults, 30% are malignant. 2 A mature teratoma is called a dermoid cyst. Dermoid cysts are benign lesions that grow slowly and can occur anywhere in the body.

## Case Presentation

**Fig. 1:**
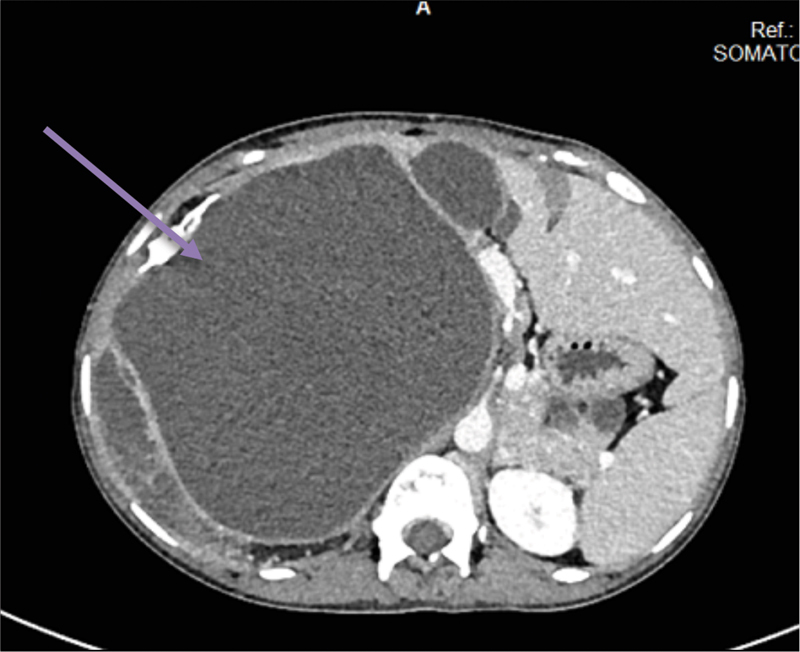
Contrast-enhanced computed tomography abdomen showing a large right-sided retroperitoneal mass compressing the right kidney and liver.

**Fig. 2:**
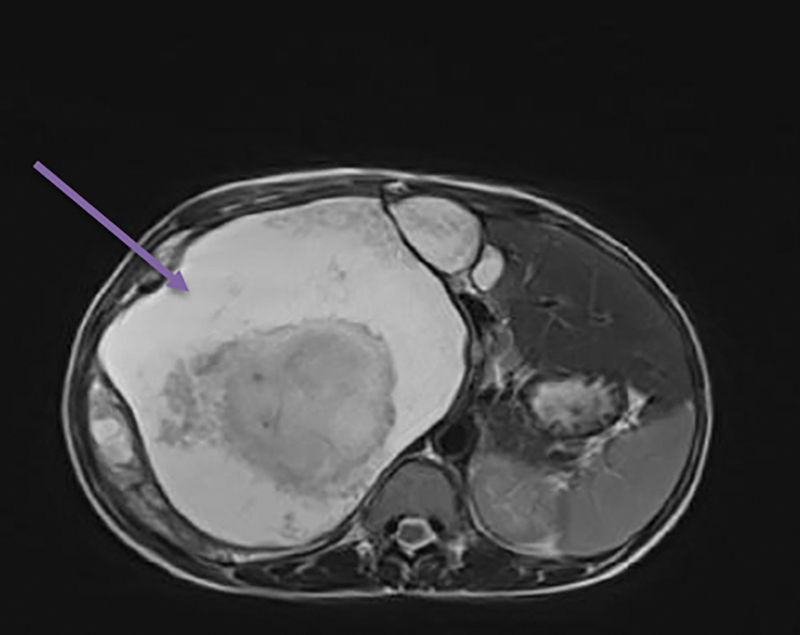
Magnetic resonance imaging cut section showing a large right retroperitoneal mass with variable tissue appearance.

**Fig. 3:**
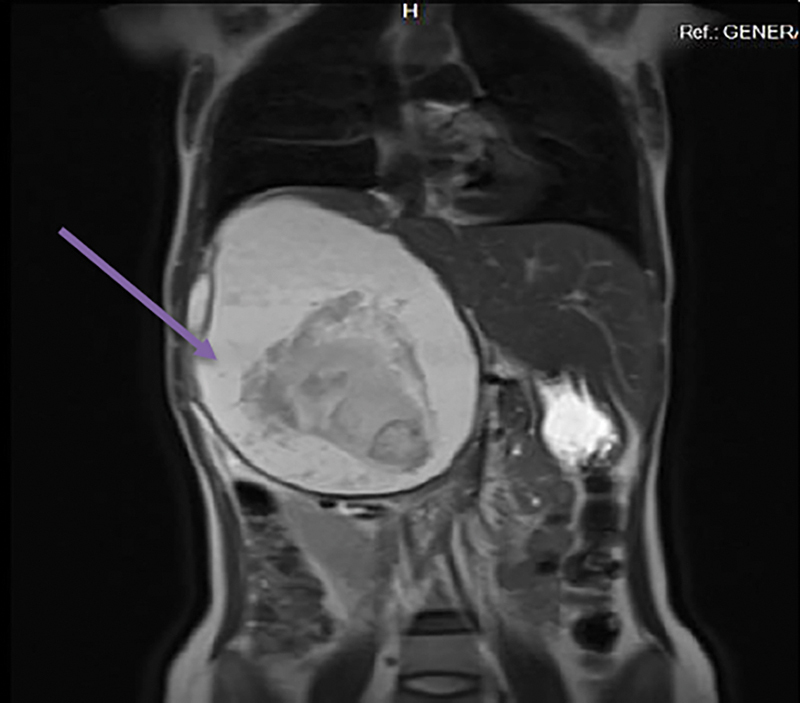
Magnetic resonance imaging coronal section showing a large right hypochondrial mass pushing the diaphragm and indenting on the liver surface along with a variable tissue appearance inside.

**Fig. 4:**
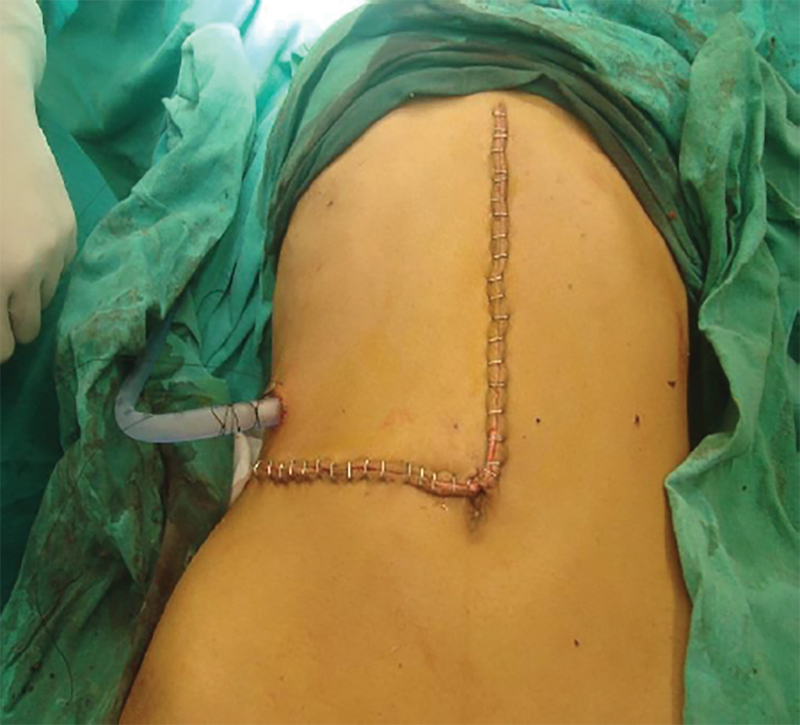
Postoperative photograph showing modified midline incision to allow for better exposure.

**Fig. 5:**
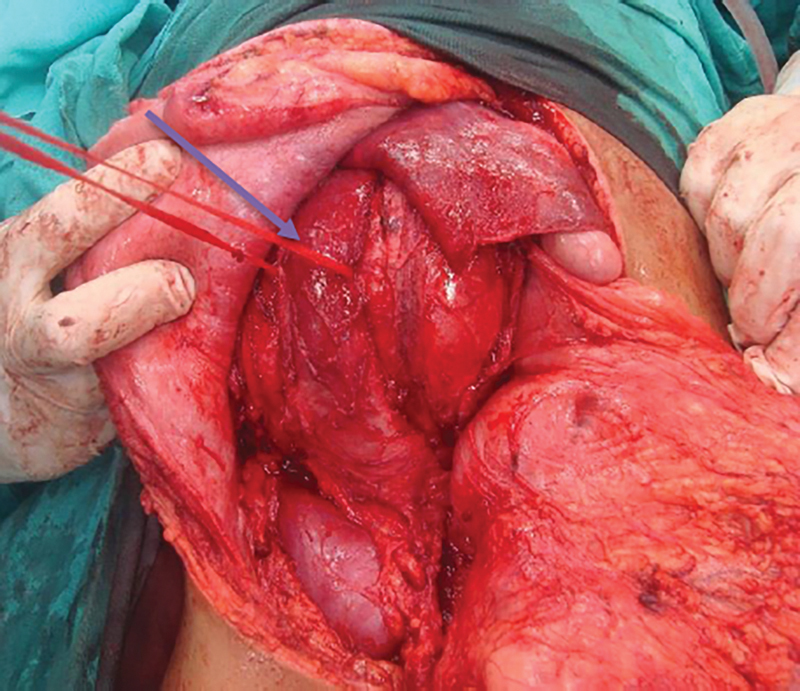
Intraoperative photograph showing right hypochondrial lesion compressing the liver, posteriorly kidney, and anteriorly the vena cava, which has been flattened out by the mass (arrow).

**Fig. 6:**
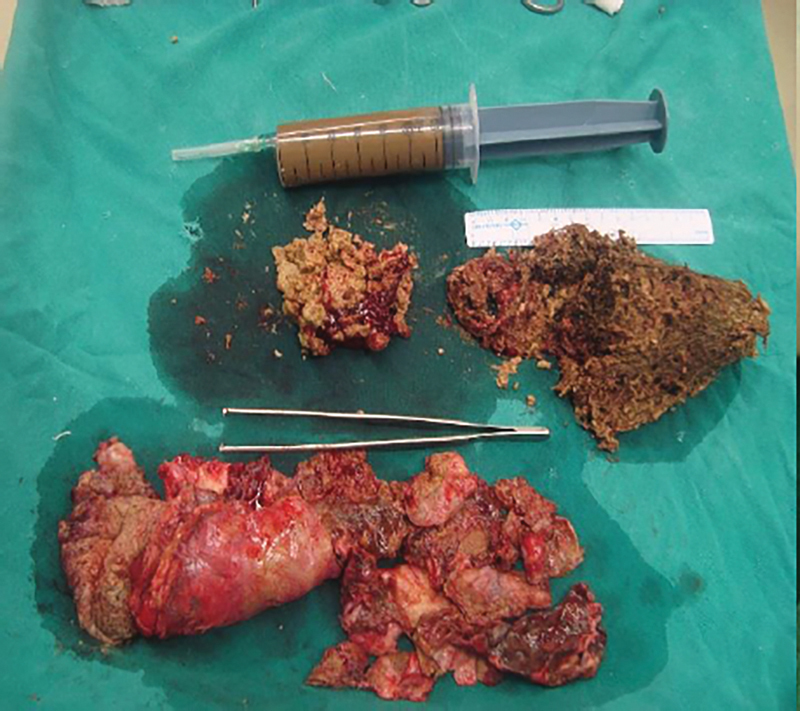
Cut section of cyst showing mature tissues, hairs, and cystic fluid.

A 20-year-old female patient presented with chief complaints of swelling in the right upper abdomen since childhood and back pain for the past 4 years. The swelling progressed in size with time and for the past 2 months, she noticed a rapid increase in size. The swelling was associated with dull aching back pain, more pronounced in the past 4 years. There was no associated history of any gastrointestinal symptoms. Per abdomen, examination revealed a nonmobile lump of around 15 cm in size in the right hypochondrial region extending to the epigastric region. All routine laboratory investigations were unremarkable. Ultrasonography showed a large heterogeneous mass with multiple internal septations and areas of fat within it in the right hypochondrium and the mass was displacing the inferior vena cava anteriorly. The features were suggestive of retroperitoneal dermoid. Upper gastrointestinal endoscopy was unremarkable. Contrast-enhanced computed tomography (CECT) abdomen revealed a 14.3 × 14.1 × 17.4 cm well-defined, heterogeneously hypoattenuating, nonenhancing complex cystic mass with focal areas of calcifications and fat attenuation in retroperitoneum ( Fig. 1 ). The lesion was causing compression and displacement of inferior vena cava anteriorly. Superiorly mass was abutting segment V and VI of the liver and inferiorly compressing the right kidney. Anteromedially, it was causing compression and displacement of the portal vein, pancreas, and small bowel. Features were suggestive of retroperitoneal/retrocaval dermoid. Magnetic resonance imaging (MRI) abdomen ( Fig. 2 ) also revealed a large 14.3 × 14.1 × 17.4 cm well-defined, multiloculated heterogeneous cystic mass with multiple internal septations in retroperitoneum on right side inferior to the liver. The cystic component was hyperintense on T1 and T2 and showing no suppression on Fat-Sat sequences ( Fig. 3 ). Medially, it was abutting the right psoas muscle and corresponding vertebrae; however, no extension into the spinal cord was noted. Features were suggestive of retroperitoneal/retrocaval dermoid. The patient was taken up for exploratory laparotomy using a modified midline incision angling toward the right side in the lower part for better access ( Fig. 4 ). A large tumor was found behind the inferior vena cava causing it to be flattened out over the swelling ( Fig. 5 ). The tumor was mobilized circumferentially and excised, taking a due care to avoid any untoward trauma to nearby structures or breach of the cyst wall. The abdominal cavity was examined for any other abnormality or residual cysts. No other abnormality was detected. The postoperative stay was uneventful. Histopathological examination report revealed that the cut surface was gray-brown to gray-white filled with pultaceous material, hair, and teeth. Rokitansky protuberance and few fatty, bony areas were identified ( Fig. 6 ). Maximum wall thickness ranged from 1 to 2 cm. Microscopic examination showed multiple sections of the wall showing cyst lined by stratified squamous epithelium with the presence of keratinization at places and ciliated to columnar lining at other places with the presence of hair follicles and pilosebaceous unit. Also, bundle of nerves, dilated lymphatics, adipose tissue, and islands of cartilage were seen. Focal areas show nests of cells with neuroendocrine differentiation, adrenal tissue, gastric mucosa and glands, and liver parenchyma. No immature component was identified. Features were suggestive of mature cystic teratoma. There was no evidence of any recurrence in the follow-up. No specific gastrointestinal symptoms were seen.

## Discussion

Primary retroperitoneal masses can be classified as benign or malignant or as solid and cystic. Benign tumors account for around 20% of retroperitoneal tumors and include lipoma, neurofibroma, neurilemmoma, leiomyoma, extra-adrenal chromaffinomas, paraganglioma, mucinous cystadenoma, and hamangiopericytoma. Malignant tumors account for around 80% of retroperitoneal tumors and include liposarcoma, leiomyosarcoma (50%), lymphoma (commonly non-Hodgkin lymphoma), malignant tumors from specific organs, germ cell tumors, chordomas, and Retroperitoneal Lymph Node (RPLN) secondaries with hard nodules. 3 Cystic masses can be classified as neoplastic and non-neoplastic. Neoplastic cystic masses include mature teratomas, mucinous cystadenomas, and cystic mesotheliomas. Non-neoplastic cystic masses include lymphangiomas, lymphoceles, urinomas, hematomas, Mullerian cysts, epidermoid cysts, and pancreatic pseudocysts. Fat-containing retroperitoneal masses are teratoma, lipoma, and liposarcoma (well differentiated/dedifferentiated). Retroperitoneal masses with calcification include paraganglioma, ganglioneuroma, malignant fibrous histiocytoma, and dedifferentiated liposarcoma. 3

Teratomas belong to a variety of nonseminomatous germ cell tumors and are therefore usually found within the testis or ovary. 4 However, they may also arise from nests of germ cells that have been left behind during embryonic migration of germ cells from the posterior dorsal ridges. Therefore, they can be found, usually in the midline, from the pineal, base of skull, mediastinum, and retroperitoneum to the sacrococcygeal region. 2 These tumors are characterized by the presence of mature tissues derived from all three embryonic germ layers. 5 6 The most common tissues are ectodermal (skin, hair, teeth, etc.), although endodermal (intestinal, respiratory epithelium) and mesodermal (fat, muscle) tissues are also present. 2 Teratomas having the presence of more undifferentiated tissues are more malignant, while those with more differentiated tissues are more benign. 7 They often contain both cystic and solid components. 8 Teratomas can, therefore, be further classified according to the number of layers present (monodermal, bidermal, and tridermal), according to the epithelial lining (epidermoid, dermoid and teratoid), according to the degree of differentiation (mature and immature), according to content (solid, cystic or mixed), and according to presence of malignancy (present or not). 3

Mature cystic teratomas are known as dermoid cysts and are composed of tissues from at least two of the three germ layers. They commonly present as back pain (due to compression of muscles, vertebral column) or nausea, vomiting, constipation/obstipation, colicky pain, urinary retention, hypertension, mesenteric ischemia, intermittent claudication, renal insufficiency, pedal edema, tingling, numbness, and weakness of lower limbs. 8 9 It is usually large, nonmobile and deeply placed. Confirmation is by ultrasound, CT scan, and MRI. A CT-guided biopsy can be undertaken to confirm the diagnosis. CECT is the investigation of choice for distinguishing between different types of retroperitoneal masses. 7 Surgical management is the key with complete surgical excision as the goal. Laparoscopy is the preferred method in small lesions with good planes. For larger lesions, an open approach is preferred. Tumor markers like alpha-fetoprotein and human chorionic gonadotrophin are important in the follow-up management of malignant lesions.

## Conclusion

Retroperitoneal dermoid is a rare presentation and is usually not detected due to its small size and asymptomatic nature. When large, it presents as a nonmobile mass, associated with back pain and other symptoms due to compression of adjacent organs. CECT is a very useful modality in diagnosing the retroperitoneal dermoids as well as in planning surgery. Complete enucleation is the surgical goal except malignancy where it may be difficult due to metastasis. Follow-up is warranted when malignancy is confirmed with regular CT scans and tumor marker levels.
